# Optimization of the optical coupling in nanowire-based integrated photonic platforms by FDTD simulation

**DOI:** 10.3762/bjnano.9.209

**Published:** 2018-08-22

**Authors:** Nan Guan, Andrey Babichev, Martin Foldyna, Dmitry Denisov, François H Julien, Maria Tchernycheva

**Affiliations:** 1 Centre de Nanosciences et de Nanotechnologies (C2N), UMR9001 CNRS, University Paris-Sud, University Paris-Saclay, 91405 Orsay, France; 2 ITMO University, Kronverkskiy Prospekt 49, 197101 St. Petersburg, Russia; 3 LPICM-CNRS, Ecole Polytechnique, Université Paris-Saclay, 91128 Palaiseau, France,; 4 Saint Petersburg Electrotechnical University "LETI", ul. Professora Popova 5, 197376 Saint Petersburg, Russia

**Keywords:** FDTD modeling, nanowire LED, nitride nanowires, photonic integrated circuit, photonic platform, SiN/InGaN co-integration, visible light communication

## Abstract

The optimized design of a photonic platform based on a nanowire light emitting diode (LED) and a nanowire photodetector connected with a waveguide is proposed. The light coupling efficiency from the LED to the detector is optimized as a function of the geometrical parameters of the system using the finite difference time domain simulation tool Lumerical. Starting from a design reported in the literature with a coupling efficiency of only 8.7%, we propose an optimized photonic platform with efficiency reaching 65.5%.

## Introduction

Today, infrared (IR) photonic integrated circuits (PICs) represent a well-established technology with numerous applications in optical telecommunications [1–4]. However, for life science applications (biosensors, molecular diagnostics, food inspection, etc.) visible light communication systems are required [5–6]. The technology for visible wavelength PICs is much less mature than for IR PICs, and different approaches have been explored. One exploratory approach for visible photonics consists of the monolithic integration of InGaN emitters/detectors with GaN-based waveguides [7–9]. This approach presents the advantage of providing both passive and active elements, however it brings many fabrication challenges (such as the necessity of low-loss GaN waveguides, etc). For passive photonic circuits used in the visible light range, SiN circuitry is gaining increasing attention [6,10]. SiN elements can be embedded into SiO_2_ [10], or alternatively, SiN suspended membranes can be used to enhance the performance [11–13]. However, for both approaches, to generate and detect light, active elements need to be co-integrated with the SiN platform.

Thanks to their direct tunable bandgap [14] and reduced dislocation density [15–16], nitride nanowires (NWs) have become important materials for optical components in the visible to ultraviolet (UV) spectral range [17–18]. Despite their small size, single NW light emitting diodes (LEDs) can produce bright electroluminescence [19]. Single NW photodetectors based on the photoconductive operation principle [20] or p–n junction photodiodes [21] have demonstrated high sensitivity. For these reasons, single NW components are considered as promising building blocks for integration with SiN photonic platforms. For the wafer-scale fabrication, NWs can be assembled by di-electrophoresis [22]. Compared to the conventional nitride photonic integrated circuits, NW-based PICs can offer higher efficiency, lower energy consumption and larger design freedom.

In addition to pure photonic systems, hybrid integrated platforms combining photonic and non-photonic elements are highly desired for various applications in bio- or chemical sensing [23–27] or in optogenetics. For example, NWs can play the role of active elements to generate or detect the visible light on a microfluidic chip. These potential applications have motivated extensive research on NW integrated platforms [28–30].

The first important step is the on-chip manipulation of light, which can be achieved by integrating NW emitters and detectors with waveguides. Park et al. demonstrated the coupling between an electrically pumped single InGaN/GaN NW LED and a 2D photonic-crystal waveguide [28]. The authors integrated LEDs on top of a SiN*_x_* membrane photonic crystal and deposited electrical contacts. The photonic crystal waveguide was shown to efficiently guide the electroluminescence over a distance of about 20 µm. Brubaker et al. realized on-chip optical coupling between an LED and a photoconductive detector both based on GaN NWs [29]. The communication took place in free space. Using NWs located close to each other, the authors demonstrated a correlation between the LED on/off switching and the photodetector current variations. A waveguide for light channeling is expected to improve the LED–detector coupling efficiency. Recently, an integrated photonic platform consisting of an InGaN/GaN NW LED and a p–n NW photodiode interconnected with a SiN*_x_* waveguide was demonstrated [30]. The communication took place in the visible spectral range (λ ≈ 400 nm). This platform combined all the basic building blocks of the optical circuit: emitter, coupling waveguide, and photodetector. However, the waveguide was not optimized. The platform used a multimode SiN*_x_* beam with a weak coupling to the active components. The reported coupling yield between the LED and the photodiode was only 8.7%. Despite this low coupling efficiency, the communication between the components was demonstrated.

To make a viable NW photonic platform, the coupling efficiency needs to be strongly improved. The optimization of the light coupling should account for technological constraints related to the typical NW dimensions and fabrication procedures. In this paper, we theoretically analyze the light propagation between a NW emitter and a detector coupled with a SiN*_x_* waveguide. Using finite difference time domain (FDTD) simulations, we propose an optimized waveguide design, for which 65.5% of the emitted light is coupled into the NW photodiode.

## Simulation

**Figure 1:**
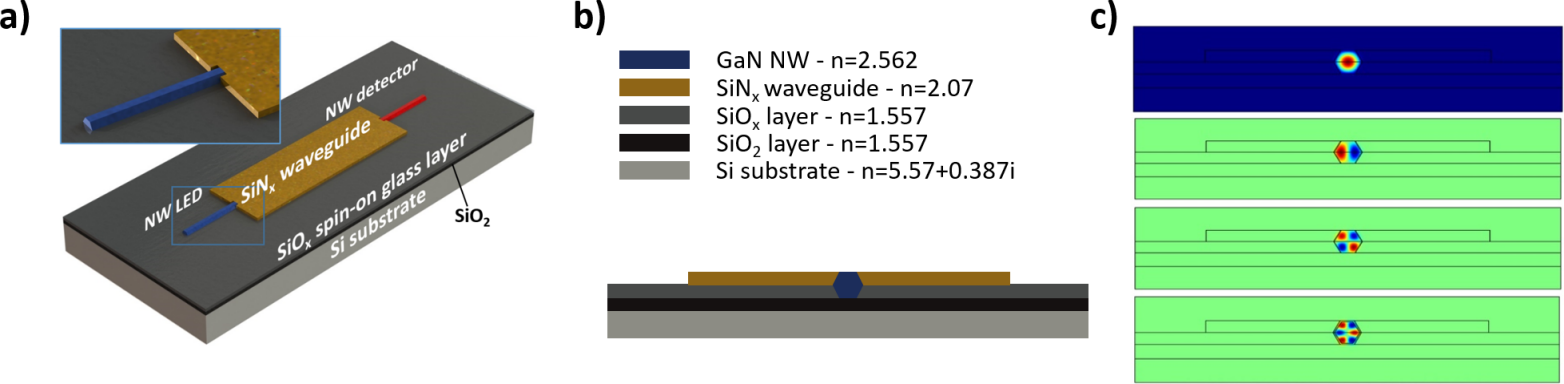
(a) Schematic illustration of the platform geometry used for the FDTD simulations. The platform consists of a NW LED, waveguide and NW photodetector; (b) Front-view cross-section schematic illustration with refractive indices for materials used in the simulations given; (c) The fundamental mode and three higher order modes of a hexagonal GaN NW with a diameter of 1 µm for the wavelength of 400 nm. The fundamental mode was used for the FDTD simulations.

For our optical simulations, we chose a platform architecture that follows the experimental realization as previously described [30]. The considered geometry is illustrated in Figure 1a,b. The horizontal NW LED with a hexagonal cross-section has a diameter of 1 µm. It is positioned on a SiO_2_ layer on top of a Si substrate. The thickness of the SiO_2_ layer is varied to find the minimum thickness necessary to avoid light coupling from the platform components to the absorbing Si substrate. Following the experimental realization of [30], an encapsulating spin-on-glass layer partially covering the NW is considered (used as a mechanical support for the contacts). The NW LED is buried to one half of its diameter into this spin-on-glass (SiO*_x_*) layer, and the SiN*_x_* waveguide is positioned on top of the spin-on glass. In the optimization, calculations without the spin-on-glass layer are also performed. The LED is connected to a straight SiN*_x_* waveguide, which has a thickness chosen to match the top surface of the guide with the top facet of the lying hexagonal NW. The end of the NW overlaps with the waveguide over a 1.5 µm long segment. On the opposite end of the waveguide, a horizontal NW detector is positioned, with its 1.5 µm long end overlapped with the waveguide, and it is also embedded into a spin-on-glass layer. The axes of the LED and detector NWs are aligned with the waveguide direction as shown in Figure 1a. The optical refractive indices for materials used in the simulations are: *n* (GaN) = 2.562, *n* (SiO_2_) = 1.557, *n* (Si) = 5.57 + i0.387, *n* (SiN*_x_*) = 2.07. The spin-on-glass SiO*_x_* was assumed to have a refractive index of *n* (SiO*_x_*) = 1.557. The presence of the InGaN quantum wells in the NW is neglected due to its nanometer dimensions and small optical contrast with GaN. The length of the NW LED and the detector is 10 µm, and the waveguide between them is 31 µm long. The waveguide width is varied in this work to optimize the optical coupling.

Both two-dimensional (2D) and three-dimensional (3D) optical models were applied using multiphysics Lumerical software [31], which numerically solves the light propagation equations using the FDTD method. 2D/3D Maxwell equations are solved in both the time and frequency domains. Perfectly matched layers were used as the boundaries to truncate computational regions. The computational regions cover all the volume of the functional elements with a margin of ≈5 µm. For 3D simulations, the minimum mesh cells per wavelength were set as 10. For 2D simulations, an auto non-uniform mesh type was used with mesh accuracy level 2. Considering the computational capability, the minimum mesh step is fixed at 0.25 nm for both 2D and 3D simulations, giving an accuracy sufficient for this study. As a reference, with the above setting, the 3D simulation of the same geometry as used in [30] required a memory of 6 GB for 107 million FDTD Yee nodes. For the 2D simulation at the horizontal plane in the middle of the waveguide, a memory of 500 MB was required for 2.1 million FDTD Yee nodes.

We define the coupling efficiency as the ratio between the light intensity leaving the NW detector and the light intensity entering the NW LED (both integrated over the hexagonal NW cross-sections of 1.5 µm diameter before the end facet of the LED and 1.5 µm after the entrance facet of the detector, respectively). Note that all the transmission values in this paper are calculated automatically by Lumerical software, defined by the normalized integration of the Poynting vector over a defined surface. The coupling efficiency was optimized by varying the geometrical parameters of the platform. Considering the computational capacity, 2D simulations at the vertical cross-sectional plane in the middle of the waveguide were used to evaluate the impact of the SiO_2_ spacer thickness and to study the influence of the spin-on-glass SiO*_x_* layer by removing it. 2D simulations at the horizontal cross-sectional plane in the middle of the waveguide were performed on the system without spin-on glass encapsulation to find the optimal width of the SiN*_x_* waveguide. For a final coupling efficiency calculations, 3D simulations were applied to the geometry optimized using 2D simulations and also to the initial geometry from [30].

We note that for the typical NW dimensions used in experiments, the LED and the photodetector NWs themselves behave as multimode waveguides. Figure 1c illustrates the fundamental and several higher order modes in a GaN nanowire with hexagonal shape calculated using COMSOL [32] mode solver at a wavelength of 400 nm. It is difficult to estimate precisely which modes are excited in an operating core/shell LED. For light propagation simulations, we simulate the light generation in the LED nanowire by injecting the fundamental mode in the nanowire 3 µm away from the SiN*_x_* waveguide input facet.

## Results and Discussion

First, the 3D FDTD simulation of the LED/photodetector platform with a geometry exactly reproducing the experimental realization of [30] was performed. The thickness of the SiO_2_ layer beneath the NW was 600 nm. The thickness of the spin-on encapsulating layer was 500 nm, and the SiN*_x_* waveguide thickness was 500 nm with the width of 10 µm. For these geometrical parameters, separate COMSOL simulations show that the SiN*_x_* waveguide sustains a large number of modes (in particular, many modes with the same vertical profile and a various number of lobes in the lateral direction are present).

**Figure 2:**
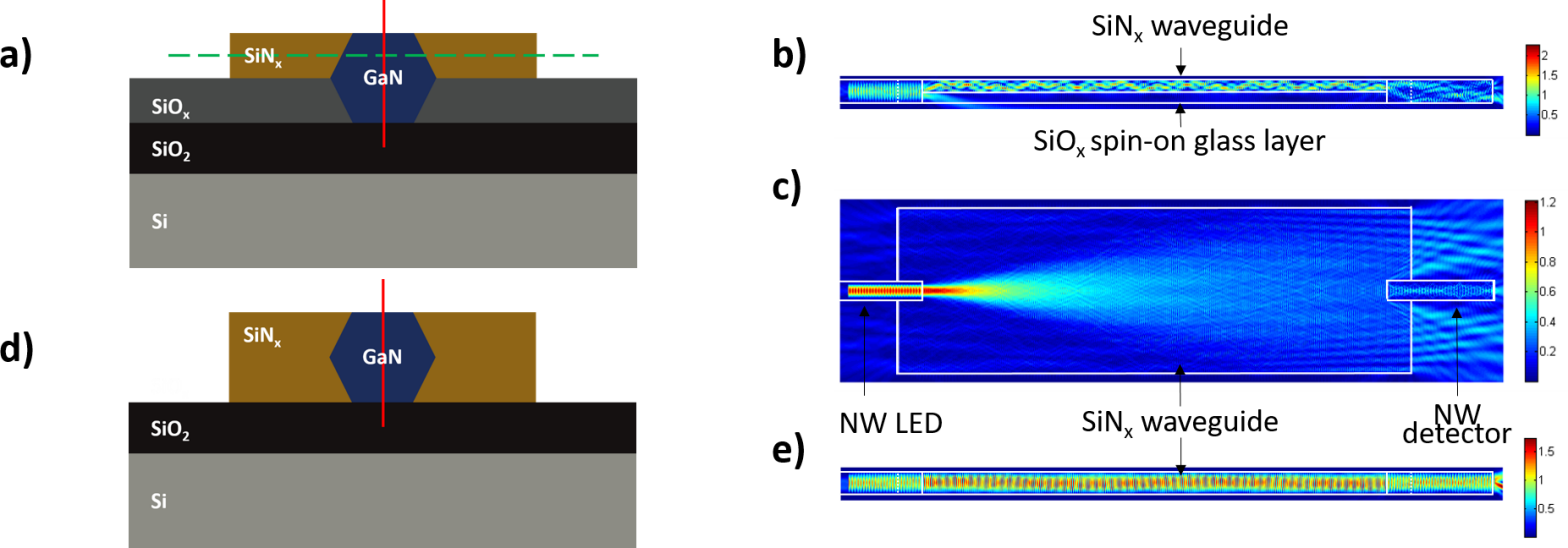
(a) Front-view cross-section schematic showing the cutting positions for 2D simulated cross-sections in panels (b) and (c); Electric field distributions for a (b) vertical and (c) horizontal cross-section taken at the middle of the waveguide (cutting position corresponding to the solid red line and dashed green line in (a)). Waveguide width is 10 µm. (d) Front-view cross-section schematic of the platform without spin-on glass encapsulating layer showing the cutting position for cross-section of panel (e). (e) Influence of the spin-on-glass layer: electric field distributions in the vertical cross-section layer in the middle of the waveguide without spin-on glass encapsulating layer (cutting position corresponding to red solid line in panel (d)).

The simulated electric field distributions in the horizontal and vertical cross-sections in the middle of the waveguide are shown in Figure 2. The LED-waveguide coupling yield, which is defined as the ratio between the light intensity integrated over the waveguide cross-section after propagation over 6 µm in the waveguide and the intensity in the NW LED 1.5 µm away from the entrance of the waveguide, is 55%. Almost half of the light is lost during the coupling from the NW LED to the waveguide. This loss is not due to the reflection at the NW/waveguide interface, but, as shown in Figure 2b, it originates mainly from the partial overlap in the vertical direction between the NW and the waveguide due to the spin-on-glass layer beneath the SiN*_x_*. An attenuation of the light intensity of 3% is observed along the light propagation path in the waveguide from 6 µm to 26 µm. This attenuation is due to the divergence of the light exiting the NW LED (as shown in Figure 2c), which is not guided but coupled to free space modes at rather large angles. The numerical aperture (NA) of the waveguide is 1.81, calculated by the formula


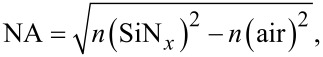


corresponding to a maximal total output angle of 122°. Considering the coupling from the waveguide to the NW detector, a strong loss is observed due to the large width of the waveguide, which leads to the leakage of light into free space. This loss can be clearly seen in Figure 2c. The coupling efficiency from the waveguide to the detector is 14%. As a result, a coupling yield of the entire system (i.e., from the LED to the detector) of only 8.7% is achieved, which is not sufficient for the previously discussed applications.

This simulation of the experimental system from [30] allowed the origin of the low coupling efficiency to be identified, namely the poor vertical overlap between the NWs and the SiN*_x_* waveguide due to the spin-on-glass encapsulation and the poor coupling from the wide SiN*_x_* waveguide to the NW photodetector. Therefore, we have focused on the optimization of the LED-to-waveguide coupling and then on the waveguide-to-photodetector coupling to improve the design of future devices.

As shown in Figure 2b, the coupling loss from the LED to the waveguide is mainly caused by a weak overlap between the NW modes and the waveguide. To decrease this loss, the spin-on-glass layer beneath the SiN*_x_* waveguide should be removed so that the waveguide has a thickness equal to the NW diameter (as shown in Figure 2d). The vertical cross-sectional 2D simulation of the platform without the spin-on-glass layer shows a significant efficiency improvement of the first coupling, which increases from 55% to 77.1%. The electric field distribution in the vertical cross-section in the middle of the waveguide is shown in Figure 2e. We note that this change of the architecture is feasible for the platform fabrication: the spin-on-glass supporting layer can be removed by dry etching after the metallic contact deposition without any damage of the device, and the SiN*_x_* waveguide can be fabricated after this etching step.

Taking into account that the Si substrate is strongly absorbing at 400 nm, a simulation with different thicknesses of the SiO_2_ layer was done to evaluate the impact of the SiO_2_ spacer thickness on the losses due to the light coupling and absorption in Si. The results demonstrate that for a SiO_2_ thickness larger than 300 nm, the absorption of the Si substrate becomes negligible due to total internal reflection at the GaN/SiO_2_ interface and the transmission of the waveguide is independent of this thickness. This means that any thickness more than 300 nm efficiently decouples the optical platform from the underlying substrate. In the following, the 600 nm value was fixed (to be comparable with the previous simulation).

A large number of in-plane modes in the SiN*_x_* waveguide results in poor matching with the NW photodetector. A significant portion of the light couples from the waveguide to free space modes. To increase the coupling yield, the waveguide width should be adjusted to couple light strongly also in the lateral direction. For this optimization, the spin-on-glass encapsulating layer was omitted, the waveguide height was fixed at 1 µm, and the FDTD simulations were performed supposing a rectangular waveguide with variable width. To reduce the computation time, a 2D simulation was performed at a horizontal cross-section plane in the middle of the waveguide. Although the 3D simulation is more rigorous than the 2D one, the 2D simulation leads to acceptable accuracy for this specific optimization of width for reducing light dispersion in the lateral direction.

**Figure 3:**
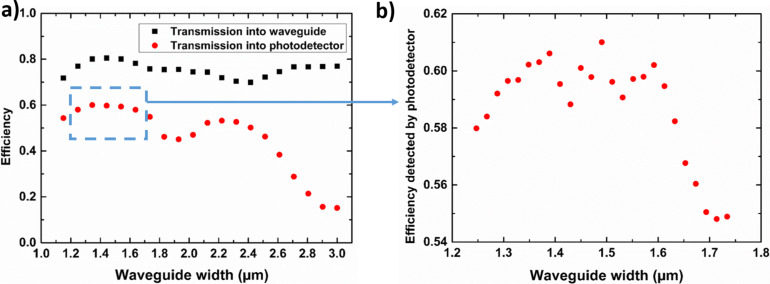
(a) Coupling efficiency from the LED to the waveguide (black squares) and from the LED to the detector (red circles) as a function of the waveguide width calculated with a step of 0.1 µm. (b) Coupling efficiency from the LED to the detector as a function of the waveguide width around the value of 1.5 µm calculated with a step of 22 nm.

Firstly, the LED-waveguide coupling yield as a function of the waveguide width was studied. The optimization with a rough step for the width showed that for a waveguide larger than 3 µm, there is a strong leakage in the waveguide-to-detector coupling. For a width smaller than 1 µm, i.e., smaller than the NW diameter, the coupling efficiency from the LED to the waveguide also degrades. We have refined the simulation between these two values taking a smaller step of 0.1 µm. As shown in Figure 3a, from 70% to 80% of the light is transmitted into the waveguide from the NW LED for this range of widths. This means that the LED-waveguide coupling efficiency does not vary much when the waveguide is wider than the NW LED diameter, as expected.

Next, the total coupling efficiency from the LED to the photodetector was simulated in a 2D configuration as a function of the waveguide width. As shown in Figure 3a, the best light coupling is achieved when the waveguide width is around 1.5 µm. With a waveguide wider than 2.5 µm, the transmission drops rapidly because of the multimode nature of the waveguide and the leakage to free space, as mentioned previously. To more precisely estimate the optimal waveguide width, we have reduced the variation step of the waveguide width to 22 nm in a range from 1.25 to 1.75 µm. We note that the control of the waveguide width with a precision of ≈20 nm is within reach of electron beam lithography processing techniques. The FDTD simulations for the waveguide widths around 1.5 µm were performed as shown in Figure 3b. A coupling efficiency above 59% is calculated for a wide range of widths between 1.3 and 1.6 µm. The observed non-monotonous, fast variation of the coupling efficiency within 2.5% is likely due to the variation of the modal structure of the waveguide and the coupling of individual modes to the detector, which is quite sensitive to the waveguide width. With a waveguide width of 1.49 µm, the maximal total transmission to the photodetector reaches 61%.

**Figure 4:**
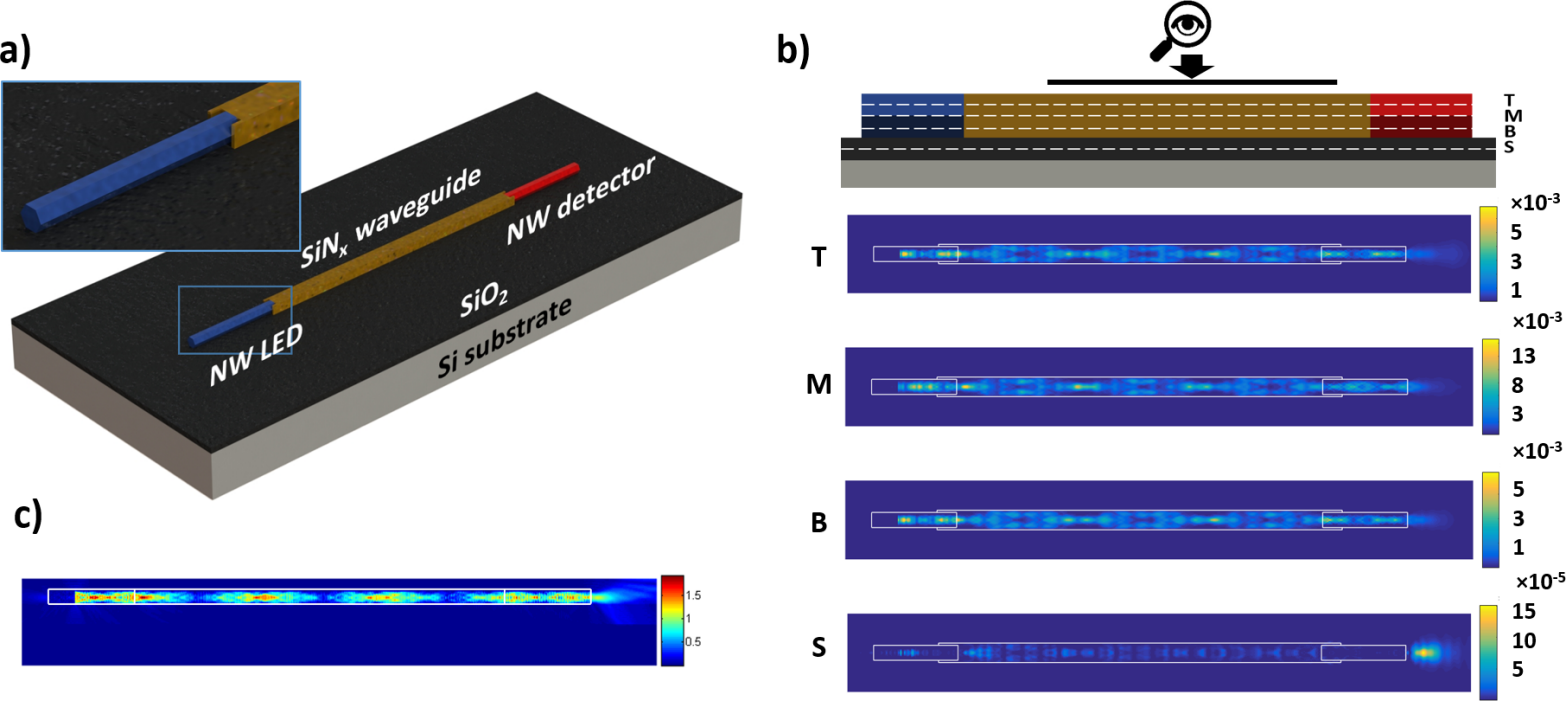
(a) Schematic illustration of the 3D FDTD simulation model consisting of a NW LED, waveguide and a NW photodetector with the optimized parameters. (b) Schematic view of layers and the top-view Poynting vector distributions (phase of the plane wave source is 0) in different horizontal layers (T, M and B: top, middle and bottom layers in the waveguide, respectively. S: horizontal layer in SiO_2_). (c) Side view of the electric field distribution in the vertical cross-sectional layer in the middle of the waveguide.

With the optimized parameters (no spin-on glass encapsulating layer, waveguide width equal to 1.49 µm), a 3D simulation was realized. In particular, the amplitude of the real part of the *y*-component (i.e., pointing along the waveguide from the LED to the detector) of the Poynting vector was analyzed. The distribution of the Poynting vector in the different horizontal layers inside the system and the electric field distribution in the vertical cross-sectional layer in the middle of the waveguide are shown in Figure 4. It should be noted that the distributions in Figure 4 show interference effects that are due to our simulation method that uses a monochromatic wave light source. These effects in our case are not physical, since we are dealing with an LED source which has significant spectral broadening and poor coherence. Nevertheless, the interference effects present in our FDTD simulations may impact the calculated transmission. We investigated the influence of the interference on the transmission by changing the phase of the wave and found out that the transmission and the Poynting vector distributions are almost independent from the phase and that these variations can be neglected (more details are given in Supporting Information File 1).

The SiN*_x_* waveguide shows efficient vertical and horizontal confinement of the propagating light. By comparing Figure 2b and Figure 4c, a significant improvement at the LED–waveguide coupling was achieved by removing the spin-on-glass SiO*_x_* layer. As shown in Figure 4b, the leakage caused by the multimode nature at the waveguide–detector coupling is mostly reduced thanks to the lateral confinement of the waveguide with a reduced width. The final efficiency detected by the NW photodetector and calculated using the 3D model is 65.5%, which is even better than the 2D simulation result due to better confinement in the 3D structure.

## Conclusion

By using FDTD simulations, the efficiency of light coupling in a NW LED–waveguide–NW photodetector integrated photonic platform was optimized from an initial value of 8.7% up to 65.5%, which is a 7.5-fold increase from the initial structure. The optical losses due to the LED–waveguide and waveguide–photodetector coupling were reduced by removing the spin-on-glass encapsulation layer and reducing the SiN*_x_* waveguide width. For NW devices with a 1 µm diameter, the optimized efficiency is reached for a 1.49 µm wide waveguide. All of the parameters are within reach of current nanofabrication capabilities. Therefore, the optimized photonic platform with a high light coupling efficiency could be implemented. This light source–waveguide–photodetector platform is the main building block of future multifunctional integrated photonic systems for photonic and sensing applications.

## Supporting Information

File 1 Additional calculation information.
