# Supplementary material for: Optimization of the optical coupling in nanowire-based integrated photonic platforms by FDTD simulation
Source: Beilstein J Nanotechnol. 2018 Aug 22;9:2248–54. doi: 10.3762/bjnano.9.209 (PMC6122179; doi:10.3762/bjnano.9.209)
Supplement: File 1 — Additional calculation information. [file Beilstein_J_Nanotechnol-09-2248-s001.pdf]

Supporting Information

for

Optimization of the optical coupling in nanowire-based integrated photonic platforms by FDTD simulation

Nan Guan[*1], Andrey Babichev[2], Martin Foldyna[3], Dmitry Denisov[4], François H. Julien[1], and Maria Tchernycheva[1]

Address: [1]Centre de Nanosciences et de Nanotechnologies (C2N), UMR9001 CNRS, University Paris-Sud, University Paris-Saclay, 91405 Orsay, France, [2]ITMO University, Kronverkskiy Prospekt 49, 197101 St. Petersburg, Russia, [3]LPICM-CNRS, Ecole Polytechnique, Université Paris-Saclay, 91128 Palaiseau, France, and [4]Saint Petersburg Electrotechnical University "LETI", ul. Professora Popova 5, 197376 Saint Petersburg, Russia

* Corresponding author

Email: Nan Guan - nan.guan@u-psud.fr

Additional calculation information

Influence of the coherence effects on the calculated transmission

In our calculations, the light generation in a NW LED is modeled by injecting a monochromatic plane wave in the fundamental mode of the NW 3 µm away from the SiN_x waveguide input facet. This approach leads to an appearance of coherence effects (namely, interferences). However, these effects in our case are not physical, since we are dealing with an LED source which has an important spectral broadening and a poor coherence.

The crucial parameter of our optimization is the power flow injected into the nanowire detector with respect to the power emitted by the nanowire LED. This parameter called "transmission" is defined by the normalized integration of the Poynting vector over the cross-section of the nanowire detector. The transmissions are calculated by the Lumirical software with the following formula:

$$T(f) = \frac{\frac{1}{2}\int real\left(\vec{P}(f)^{Monitor}\right)\cdot d\vec{S}}{sourcepower},$$

where T(f) is the normalized transmission as a function of frequency, P(f) is the Poynting vector normal to the surface, and dS is the surface normal.

Since the above-mentioned interference effects may impact the calculated transmission, we performed simulations by changing the phase of the injected plane wave to evaluate the importance of these effects. We did the calculation of the transmission for 11 different values of the plane wave phase. The resulting transmission varied only weakly from 65.48 % to 65.54 %, as shown in Fig. S1, so the impact of coherence effects can be neglected. The Poynting vector distributions (namely, the amplitude of the real part of the y-component of the Poynting vector) for these 11 different phases do not show any notable difference neither (Fig. S2 displays 2 examples of the Poynting vector distributions at 2 different phases).

In summary, the interference effects, which are artefacts of our calculation method, do not influence the results and the conclusions of the manuscript.

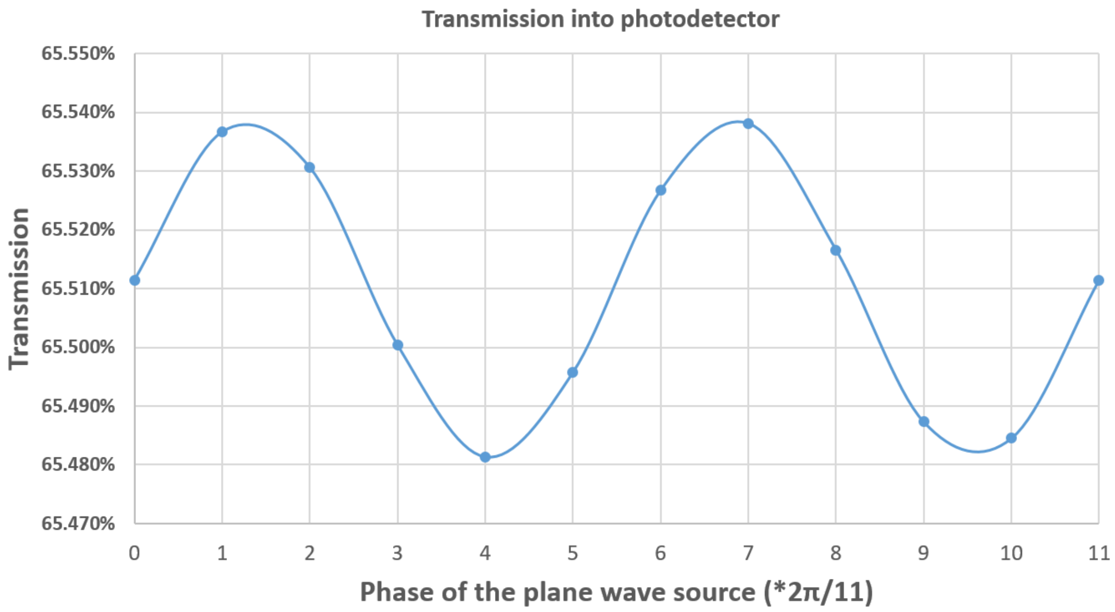

Figure S1: Transmission into the photodetector as a function of the phase of the plane wave source.

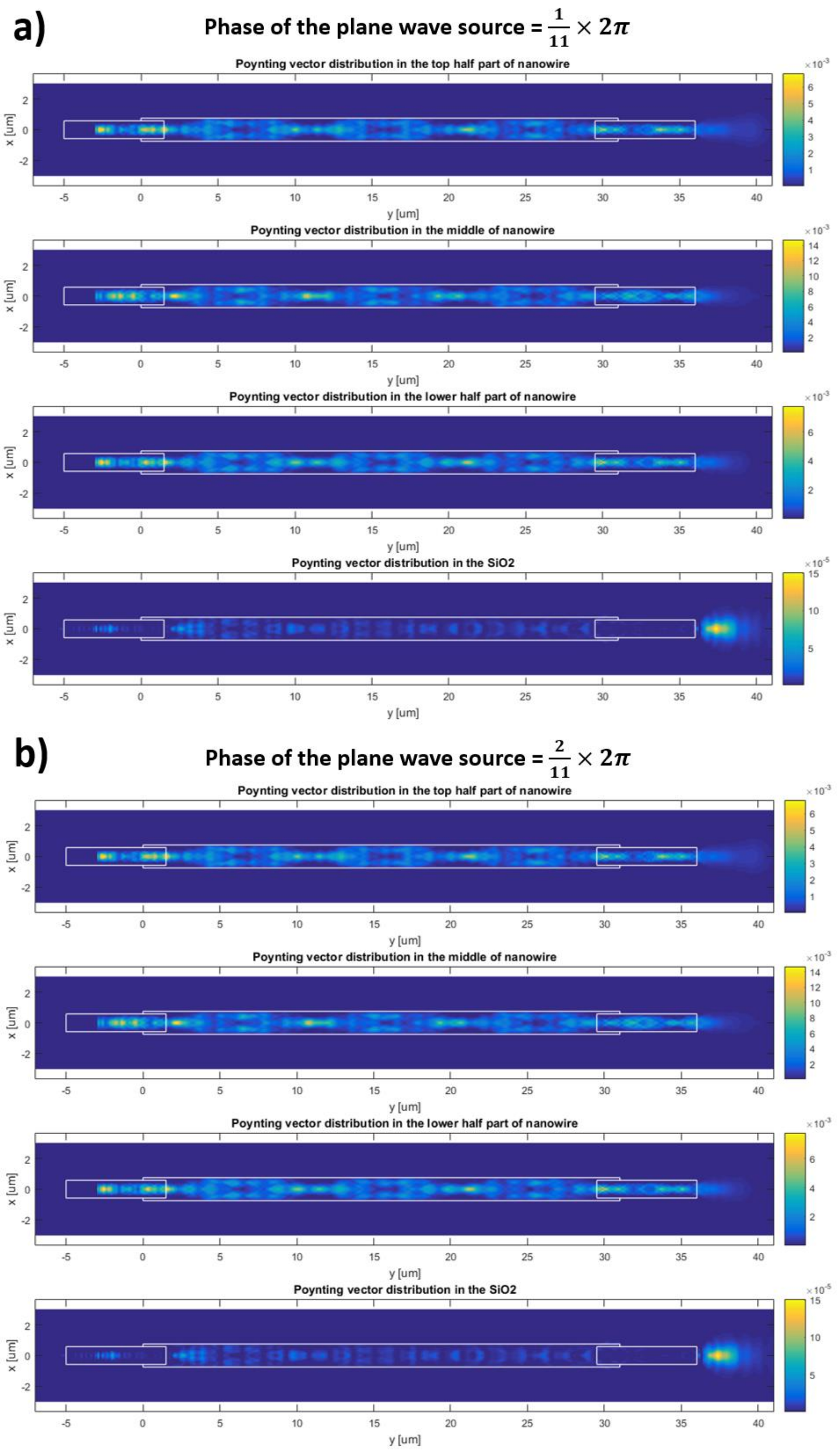

Figure S2: Top-view Poynting vector distributions in different horizontal slices with a phase of the plane wave source equal to (a) $\frac{1}{11} \times 2\pi$ and to (b) $\frac{2}{11} \times 2\pi$.
